# Prognostic value of tertiary lymphoid structures in hepatocellular carcinoma: a meta-analysis and systematic review

**DOI:** 10.3389/fimmu.2024.1390938

**Published:** 2024-06-03

**Authors:** Lingbo Hu, Xuemei Li, Changhong Yang, Baoyong Zhou, Chengyou Du, Ning Jiang

**Affiliations:** ^1^ Department of Hepatobiliary Surgery, The First Affiliated Hospital of Chongqing Medical University, Chongqing, China; ^2^ Department of Hepatopancreatobiliary Surgery, Taizhou Hospital of Zhejiang Province Affiliated to Wenzhou Medical University, Taizhou, Zhejiang, China; ^3^ Department of Hepatopancreatobiliary Surgery, Enze Hospital, Taizhou Enze Medical Center (Group), Taizhou, Zhejiang, China; ^4^ Department of Pathology, Chongqing Medical University, Chongqing, China; ^5^ Molecular Medicine Diagnostic and Testing Center, Chongqing Medical University, Chongqing, China; ^6^ Department of Pathology, The First Affiliated Hospital of Chongqing Medical University, Chongqing, China; ^7^ Department of Bioinformatics, Chongqing Medical University, Chongqing, China

**Keywords:** tertiary lymphoid structures (TLS), hepatocellular carcinoma (HCC), meta-analysis, prognosis, HBV - hepatitis B virus

## Abstract

**Background:**

Multiple investigations and scholarly articles have presented compelling evidence indicating that tertiary lymphoid structures (TLS) play a pivotal role in inhibiting and controlling the advancement of tumors. While there is an abundance of information highlighting the importance of TLS in different cancer types, their prognostic significance specifically in hepatocellular carcinoma (HCC) cancers remains unclear. Thus, this meta-analysis aimed to explore the prognostic relevance of TLS in HCC.

**Methods:**

We conducted a thorough search across four databases, namely Web of Science, PubMed, Embase, and the Cochrane Library, to identify pertinent studies. The search utilized the keywords “tertiary lymphoid structures” and “hepatocellular carcinoma.” The primary outcomes of interest encompassed overall survival (OS), recurrence-free survival (RFS), early recurrence, and late recurrence. The statistical effect size for these measures was expressed in terms of hazard ratios (HR).

**Results:**

Six studies were incorporated into the analysis. Among them, four studies, encompassing 6 datasets and involving 1490 patients, and three studies, comprising 5 datasets and involving 656 patients, respectively, investigated the correlation between intratumoral and peritumoral TLSs and the prognosis in HCC patients. The meta-analysis revealed that the presence of intratumoral TLSs is linked to longer RFS and reduced early recurrence (HR, 0.60; 95% CI, 0.50–0.67; p <0.001 and HR, 0.49; 95% CI, 0.36–0.65; p <0.001, respectively). However, no significant association was observed with OS and late recurrence. Sensitivity analysis demonstrated the robustness of these findings, and heterogeneities were minimal. Additionally, the meta-analysis did not detect a relationship between peritumoral TLSs and OS or RFS in HCC patients.

**Conclusion:**

The presence of intratumoral TLSs is correlated with better RFS and reduced early recurrence in HCC patients. Further investigation is warranted to elucidate the roles of peritumoral TLSs in the prognosis of HCC patients.

**Systematic review registration:**

https://www.crd.york.ac.uk/PROSPERO/#recordDetails, identifier CRD42023466793.

## Introduction

Liver cancer holds the sixth position in terms of incidence and ranks third in mortality (1). Hepatocellular carcinoma (HCC), constituting approximately 90% of liver cancer cases, is the predominant form of this malignancy (2). The considerable recurrence rate following liver resection contributes to an unfavorable prognosis in individuals with HCC (2). The immune contexture and spatial organization of immune cells emerge as critical factors influencing tumor invasion, metastasis, and ultimately impacting the prognosis of patients with malignancies (3–5).

Tertiary lymphoid structures (TLS) represent acquired ectopic lymphoid formations occurring in non-lymphoid tissues amidst chronic inflammation and cancer. Classified based on the presence of follicles and germinal centers, TLSs exhibit a multistage maturation process encompassing early TLSs (lymphoid aggregates), primary TLSs (lymphoid follicles formation without germinal center), and secondary TLSs (lymphoid follicles formation with germinal center) (6). The presence of TLSs has been linked to patient prognosis, indicating a favorable outlook in various cancer types, including breast (7), lung (8), colorectal (9), and pancreatic cancers (10). Nevertheless, the association between TLSs and prognosis in HCC patients remains contentious. While Finkin et al. conducted an analysis of clinical data from patients, validated findings in mouse models, explored potential mechanisms at protein and gene levels, and concluded that TLSs signify a poor prognosis in human HCC, subsequent studies have contradicted this, revealing a positive association between TLSs and better prognosis in HCC patients (11–14). Furthermore, the role of intra-tumoral or peritumoral TLSs appears to differ. To elucidate the relationship between TLSs and prognosis in HCC patients, we conducted this meta-analysis.

## Methods

### Search strategy

Systematic searches were conducted in four databases: Web of Science, PubMed, Embase, and the Cochrane Library on September 25, 2023. The search utilized a combination of Mesh terms and keywords, specifically focusing on “hepatocellular carcinoma” and “tertiary lymphoid structures.” The comprehensive details of the search strategy for all databases can be found in **Supplementary Material S1**.

### Inclusion criteria

The inclusion criteria were as follows: (1) articles specifically addressing the association between intra-tumoral and/or peritumoral TLSs and the prognosis in patients with hepatocellular carcinoma (HCC), and (2) studies that reported at least one of the following outcomes: overall survival (OS), recurrence-free survival (RFS), early recurrence, or late recurrence.

### Exclusion criteria

Excluded from consideration were noncomparative studies, abstracts, case reports, and reviews. Additionally, in cases where multiple studies featured overlapping patient cohorts, only the top study—determined by factors such as highest quality, largest sample size, or most recent publication—was included, with the other studies being excluded.

### Definition

According to the original studies incorporated in our analysis, intratumoral TLS+ (iTLS+) was characterized by the presence of any form of intratumoral TLS within the tumor samples. The density of peritumoral TLS (pTLS) was quantified as the number per square millimeter, with high density pTLS denoting samples surpassing a predetermined cut-off value. **Supplementary Material S2** provides further elaboration on the definitions of iTLS and pTLS as outlined in the included studies. OS was characterized as the duration from surgery to death, while RFS was characterized as the duration from surgery to tumor recurrence. Early recurrence was specified as a recurrence within two years following liver resection, whereas late recurrence was defined as a recurrence occurring two or more years after liver resection. In this context, OS and RFS were designated as the primary time-to-event outcomes.

### Quality assessment and data extraction

Two researchers independently conducted the initial quality assessment of each study and subsequent data extraction. For nonrandomized comparative trials, the Newcastle-Ottawa Scale (NOS) was utilized, with a score of up to 9 points (5 or less indicating low quality, 6–7 for medium quality, and 8 or more for high quality) for quality assessment (15). The extraction of study details from the included studies, such as the first author, year of publication, patient information, and tumor characteristics, was performed using pre-designed and standardized forms. Outcomes, including OS, RFS, early recurrence, and late recurrence, were extracted either directly from the original reports or indirectly by estimating with the Kaplan-Meier curve using Engauge Digitizer software (version 4.1) based on the approach introduced by Tierney et al. (16, 17). In cases of any disagreements between the two independent researchers, a third researcher facilitated resolution.

### Statistical analysis

We detected the relationship between liver background and tumor characteristics with iTLS. Risk ratio (RR) and 95% confidence interval (CI) values were determined using DerSimonian-Laird method. The hazard ratio (HR) and 95%CI values were determined using the inverse variance method for primary outcomes. Heterogeneity was evaluated through the Q statistic and I2, where I2 values of 25% and 50% indicated low and moderate heterogeneity, respectively. The choice of the test model was based on the level of heterogeneity, with the random-effects model applied for studies exhibiting I2 > 50% (18). Sensitivity analysis was conducted to assess the robustness of the conclusion. Funnel plots were employed to examine publication bias. Statistical significance was defined as a p-value < 0.05. All statistical analyses were performed using the R program (version 4.2.3).

## Results

### Study search and inclusion

**Figure 1:**
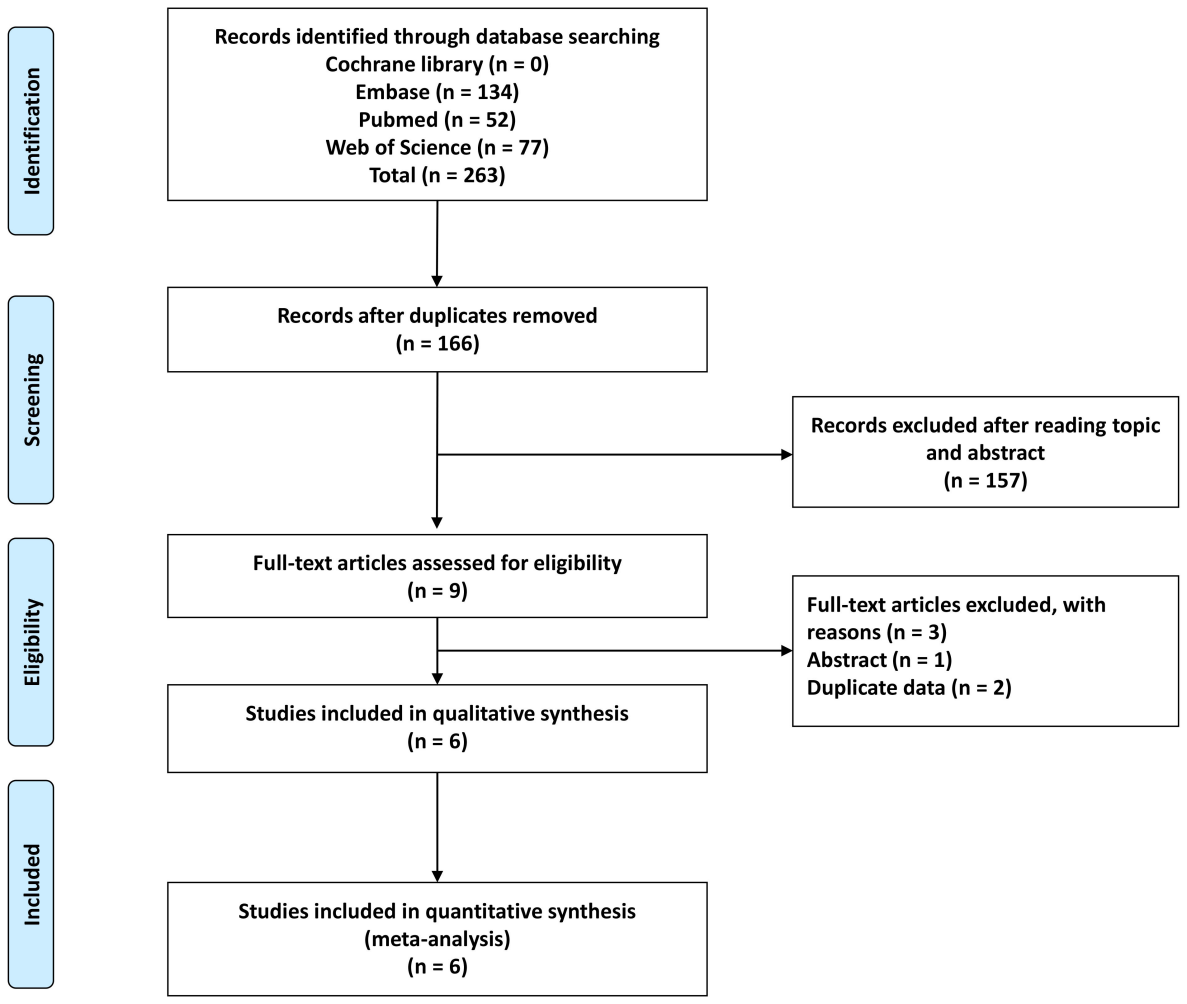
Flow chart of study selection.

A comprehensive search yielded a total of 263 articles, resulting in 166 articles after eliminating duplicates. Upon reviewing titles and abstracts, 9 records were retained. Subsequently, two studies were excluded due to duplicated data, and one study was omitted as it only presented an abstract (**Figure 1**). Consequently, this meta-analysis incorporated 6 studies (6, 11–13, 19, 20).

### Study characteristics

Four studies, comprising 6 datasets and involving 1490 patients, investigated the relationship between intratumoral TLSs and the prognosis of patients with HCC (11–13, 19). Additionally, three studies, incorporating 5 datasets and 656 patients, examined whether peritumoral TLSs are associated with the prognosis in HCC patients (6, 19, 20). Among these studies, four were conducted in China, one in France, and one utilized data from TCGA (11). The identification and counting of peritumoral and intratumoral TLS in all included literatures were achieved by immunohistochemistry. The average intratumoral TLS+ rate was 42.42%, ranging from 25.34% to 59.89%. Detailed patient and tumor characteristics can be found in **Table 1**. Only two studies reported the proportion of each stage maturation TLS, early TLSs, primary TLSs, and secondary TLSs accounted for 61.9%, 29.0% and 9.1%, respectively.

**Table 1:** Characteristics of included studies.

Study	Country	Group	Sample size	Age (years)	Gender Male/Female	HBV/HCV	Cirrhosis Y/N	AFP (ng/ml)	BCLC stage A/B+C	Tumor number (S/M)	Tumor size (cm)	Differentiation Poor/Well-Moderate	MVI Y/N
Intratumoral TLS													
Zhang 2023 (19)	China	TLS+	73	32 (>55) 41 (≤55)	62/11	48/NA	47/26	29 (>300) 44 (≤300)	NA	9/64	30 (>5) 43 (≤5)	11/62	44/29
		TLS-	97	49 (>55) 48 (≤55)	81/16	79/NA	66/31	30 (>300) 67 (≤300)	NA	16/81	61 (>5) 36 (≤5)	13/84	42/55
Jia 2022 (21)	TCGA	TLS+	218	NA	NA	NA	NA	NA	NA	NA	NA	NA	NA
		TLS-	146	NA	NA	NA	NA	NA	NA	NA	NA	NA	NA
Li 2020 (12)	China	TLS+	102	50.9 ± 13.2	91/11	91/1	70/32	42 (≥400) 60 (<400)	82/20	87/15	50 (≥5) 52 (<5)	50/52	33/69
training cohort		TLS-	201	51.3 ± 12.2	160/41	174/5	120/81	82 (≥400) 119 (< 400)	160/41	164/37	116 (≥5) 85 (<5)	77/124	72/129
Li 2020 (12)	China	TLS+	54	49.2 ± 13.5	46/8	75/1	30/24	23 (≥400) 31 (<400)	NA	NA	22 (≥5) 32 (<5)	28/26	17/37
validation cohort		TLS-	105	52.2 ± 12.1	86/19	137/2	68/37	41 (≥400) 64 (<400)	NA	NA	62 (≥5) 43 (<5)	40/65	33/72
Calderaro 2019 (13)	France	TLS+	129	76 (>60) 53 (≤60)	105/24	32/33	47/82	23 (>300) 106 (≤300)	107/22	NA	59 (>5) 70 (≤5)	22/107	56/73
HMN cohort		TLS-	144	83 (>60) 61 (≤60)	119/25	42/33	50/94	36 (>300) 108 (≤300)	110/34	NA	88 (>5) 56 (≤5)	25/119	80/64
Calderaro 2019 (13)	France	TLS+	56	9 (>60) 47 (≤60)	46/10	13/NA	50/6	23 (>300) 33 (≤300)	NA	14/42	16 (>5) 40 (≤5)	NA	NA
LCI cohort		TLS-	165	31 (>60) 134 (≤60)	145/20	43/NA	153/12	77 (>300) 88 (≤300)	NA	31/134	64 (>5) 101 (≤5)	NA	NA
Peritumoral TLS													
Zhang 2023 (19)	China	TLS high	67	32 (>55) 35 (≤55)	62/5	50/NA	47/20	26 (>300) 41 (≤300)	NA	11/56	37 (>5) 30(≤5)	7/60	32/35
		TLS low	75	34 (>55) 41 (≤55)	59/16	55/NA	45/30	25 (>300) 50 (≤300)	NA	8/67	41 (>5) 34 (≤5)	12/63	39/36
		no TLS	28	15 (>55) 13 (≤55)	22/6	22/NA	21/7	8 (>300) 20 (≤300)	NA	6/22	13 (>5) 15 (≤5)	5/23	15/13
Wen 2022 (6)	China	TLS high	61	32 (>54) 41 (≤54)	52/9	40/1	NA	29 (>37.96) 32 (≤37.96)	49/12	NA	NA	NA	NA
		TLS low	65	49 (>54) 48 (≤54)	53/12	45/1	NA	32 (>37.96) 33 (≤37.96)	42/23	NA	NA	NA	NA
Li 2021 (20)	China	TLS high	151	NA	NA	NA	NA	NA	NA	NA	NA	NA	NA
Training cohort		TLS low	89	NA	NA	NA	NA	NA	NA	NA	NA	NA	NA
Li 2021 (20)	China	TLS high	45	NA	NA	NA	NA	NA	NA	NA	NA	NA	NA
Validation cohort		TLS low	75	NA	NA	NA	NA	NA	NA	NA	NA	NA	NA

TLS, tertiary lymphoid structure; NA, not available; HBV, hepatitis virus B; HCV, hepatitis virus C; Y, yes; N, no; AFP, alphafetoprotein; BCLC, Barcelona clinical liver cancer staging; S, solitary; M, multiple; MVI, microvascular invasion.

### Quality assessment

The quality assessment details for the included studies can be found in **Supplementary Material S3**. Specifically, two studies received a score of 7 points, while another four studies received a score of 8 points. Consequently, two studies were categorized as moderate quality, and the remaining four were classified as high-quality studies.

### Intratumoral TLSs

#### Characteristics retated with iTLS

**Figure 2:**
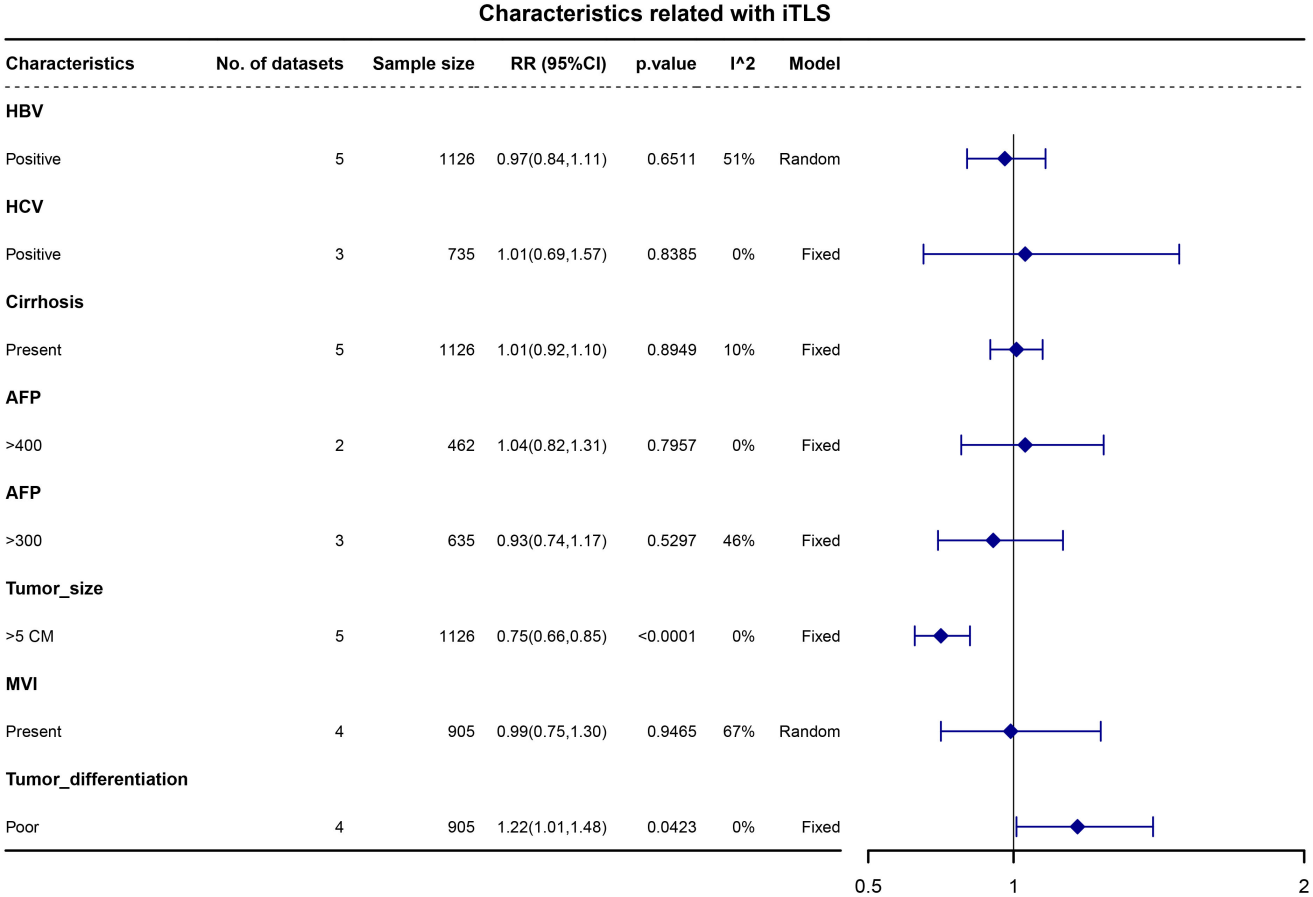
Forest plot for the relationship between intratumoral TLS+ with liver background and tumor characteristics.

We examined the associations between liver background characteristics (including HBV, HCV, and liver cirrhosis) and tumor characteristics (such as alphafetoprotein (AFP), tumor size, tumor differentiation, and microvascular invasion) with intratumoral TLS (iTLS). The pooled data revealed that patients with iTLS+ showed a higher prevalence of poor tumor differentiation (RR, 1.22; 95% CI, 1.01–1.48; p = 0.0423) and a lower prevalence of tumors larger than 5cm (RR, 0.75; 95% CI, 0.66–0.85; p <0.0001) (see **Figure 2**).

#### Outcomes

**Figure 3:**
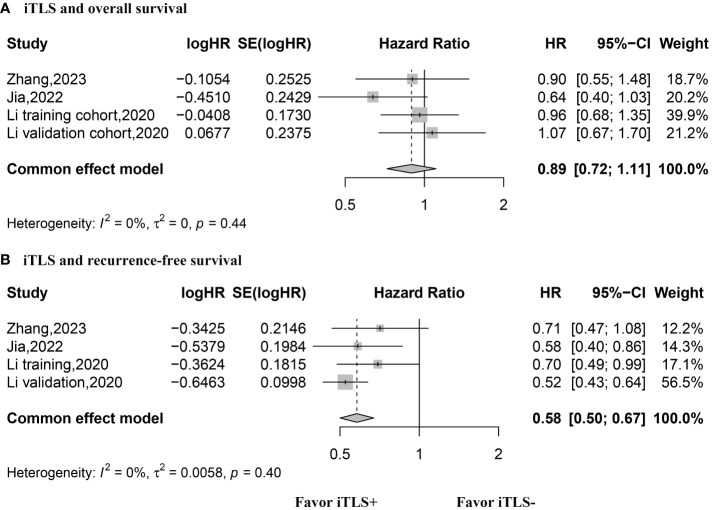
Forest plot for the relationship between intratumoral TLS+ with overall survival and recurrence-free survival. **(A)** overall survival; **(B)** recurrence-free survival.

**Figure 4:**
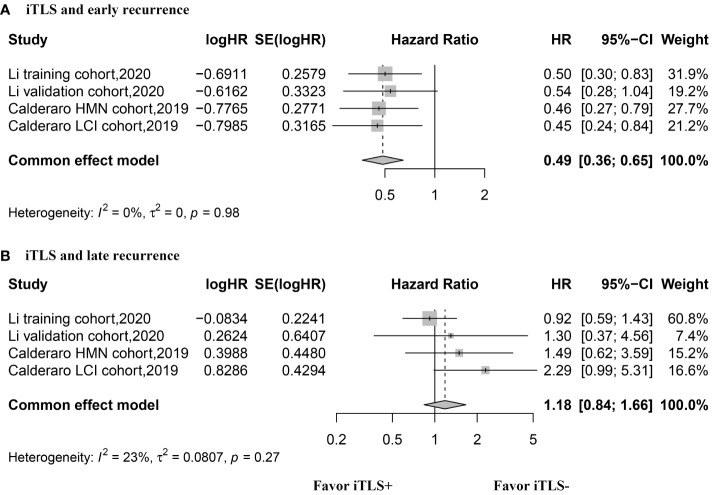
Forest plot for the relationship between intratumoral TLS+ with early and late recurrence. **(A)** early recurrence; **(B)** late recurrence.

The HR values for OS were reported in three studies, comprising 4 datasets, and were analyzed using the fixed-effects model. The aggregated data indicated that the presence of intratumoral TLSs is not correlated with OS (HR, 0.89; 95% CI, 0.72–1.11; p = 0.30) (**Figure 3**). Similarly, three studies with 4 datasets reported HR values for RFS, analyzed with the fixed-effects model. The combined data suggested that the presence of intratumoral TLS is associated with longer RFS (HR, 0.60; 95% CI, 0.50–0.67; p < 0.001) (**Figure 3**). Additionally, two studies with 4 datasets reported HR values for early recurrence, analyzed using the fixed-effects model. The pooled data revealed that the presence of intratumoral TLSs is associated with better early recurrence outcomes (HR, 0.49; 95% CI, 0.36–0.65; p < 0.001) (**Figure 4**). Furthermore, two studies with 4 datasets reported HR values for late recurrence, also analyzed with the fixed-effects model. The combined data suggested that the presence of intratumoral TLSs is not associated with late recurrence (HR, 1.18; 95% CI, 0.84–1.66; p = 0.30) (**Figure 4**).

#### Sensitivity analysis and publication bias

The sensitivity analysis demonstrated the robustness of the aforementioned results (**Supplementary Material S4**). Additionally, funnel plots of all outcome data did not exhibit noticeable asymmetry (**Supplementary Material S5**).

### Peritumoral TLSs

#### Outcomes

**Figure 5:**
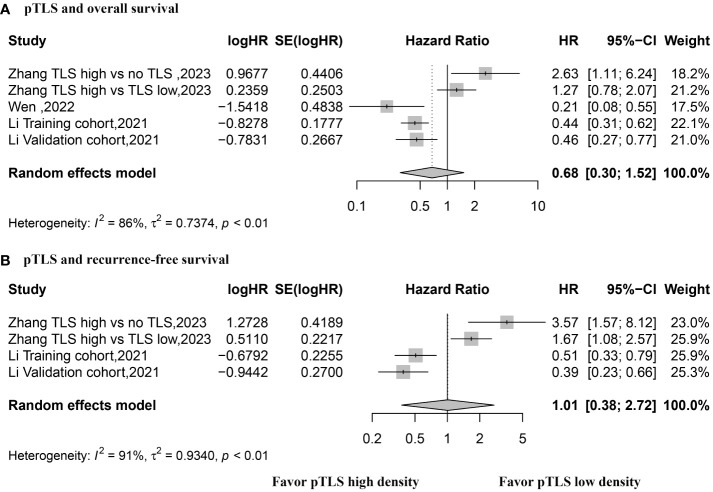
Forest plot for the relationship between high density peritumoral TLS with overall survival and recurrence-free survival. **(A)** overall survival; **(B)** recurrence-free survival.

HR values for OS were reported in three studies with a total of 5 datasets, utilizing a random effects model. The pooled data indicated that the presence of peritumoral TLSs is not correlated with OS (HR, 0.68; 95% CI, 0.30–1.52; p = 0.34) (see **Figure 5**). Similarly, HR values for RFS were reported in two studies with 4 datasets, using a random effects model. The combined data revealed no association between the presence of peritumoral TLS and RFS (HR, 1.01; 95% CI, 0.0.38–2.72; p = 0.98) (refer to **Figure 5**).

#### Sensitivity analysis and publication bias

The sensitivity analysis demonstrated the robustness of OS, while excluding one article sequentially led to a significant disparity in RFS results (**Supplementary Material S6**). As the classification criteria utilized in the study by Zhang et al. differed from those in other studies, we conducted a sensitivity analysis by excluding the data from Zhang et al. The combined data revealed that a high density of peritumoral TLSs was associated with improved overall survival (OS) and recurrence-free survival (RFS) (HR, 0.42; 95% CI, 0.32–0.55; p <0.001 and HR, 0.45; 95% CI, 0.32–0.64; p <0.001, respectively) (see **Supplementary Material S7**). Funnel plots for all outcome data did not exhibit noticeable asymmetry (**Supplementary Material S8**).

## Discussion

The results of the present meta-analysis indicate that the presence of intratumoral TLSs is correlated with improved RFS and decreased early recurrence, but no significant association was found with OS and late recurrence. Furthermore, the presence of peritumoral TLSs does not show a significant association with OS and RFS. However, the findings regarding peritumoral TLSs exhibited instability due to substantial heterogeneity.

To our knowledge, this meta-analysis is the first to investigate the correlation between intratumoral or peritumoral TLSs and the prognosis of HCC patients. The results of a robust sensitivity analysis, characterized by low heterogeneity, lend credibility to the evidence concerning the relationship between intratumoral TLSs and the prognosis of HCC patients. In contrast, the findings related to peritumoral TLSs demonstrated significant heterogeneity, and the sensitivity analysis indicated instability. Several factors must be considered to comprehend these variations. Firstly, variations in the extent of peritumoral tissue among studies were observed. Prior studies focused on intrahepatic cholangiocarcinoma noted diverse prognostic and immune-modulating roles of TLS in distinct regions (18). Zhang et al. obtained peritumoral tissue 1 cm from the tumor edge, concluding that peritumoral TLSs are associated with an unfavorable prognosis in HCC (19). Conversely, two other studies obtained peritumoral tissue 5 mm from the tumor edge, reaching the opposite conclusion (6, 20). Secondly, the proportion of TNM stage III to IV in Zhang et al.’s study was larger than that in Li et al.’s study (19, 20). Previous research indicated different prognostic roles of intratumoral TLSs in Barcelona Clinical Liver Cancer Staging (BCLC) 0-A and B-C. Thus, the varying proportion of TNM stage III to IV may be an influencing factor. Additionally, the division of peritumoral TLSs concentration is also inconsistent. Consequently, further well-designed studies, stratified by different tumor stages, distances of peritumoral tissue from the tumor edge, and suitable cut-off values for peritumoral TLSs concentration, are needed to elucidate the role of peritumoral TLSs in the prognosis of HCC patients.

According to the definition of intratumoral TLS+ provided in our paper, patients were categorized into intratumoral TLS+ and intratumoral TLS- groups, exhibiting similar overall survival (OS). However, the intratumoral TLS+ group can be further stratified into three subgroups: early TLSs+, primary TLSs+, and secondary TLSs+. In Ahn’s study, univariate Cox regression analysis identified primary TLSs+ and secondary TLSs+ as predictors of OS, while early TLSs+ were not. Furthermore, multivariate Cox regression analysis revealed that only secondary TLSs+ predicted OS (22). In our study, two included articles reported the proportions of primary TLSs+ and secondary TLSs+, accounting for 29.0% and 9.1%, respectively. The classification of intratumoral TLS+ and the low proportions of primary TLSs and secondary TLSs contribute to the lack of improvement in OS among HCC patients with intratumoral TLS+. Our findings suggest that the presence of intratumoral TLSs may enhance early recurrence but not late recurrence. This disparity in results could stem from the distinct mechanisms underlying early and late HCC recurrence. “True” recurrence, primarily arising from occult intrahepatic metastases, typically manifests as “early” recurrence and constitutes over 70% of tumor recurrences. Conversely, late recurrence is commonly associated with etiological factors and cirrhosis, which are risk factors for hepatocarcinogenesis, rather than the primary tumor itself (23). Therefore, intratumoral TLSs in primary tumors may not influence early recurrence but may affect late recurrence differently.

Lu et al. observed that higher infiltration levels of T cells in HBV-related HCCs than in non-HBV/HCV-related HCCs, which may lead to better prognosis (24). Due to the lack of sufficient raw data, we did not conduct a subgroup analysis on this topic. However, results from one of the included articles verified the above finding (12). They observed that in the HBV-positive patient group, patients with iTLS+ had better RFS and early recurrence than those with iTLS-, while in the HBV-negative patient group, patients with iTLS+ and iTLS- had similar RFS and early recurrence. These results indicate that the effects of iTLS on prognosis of HCC patients are different in viral hepatitis and non-viral environments.

Several limitations should be acknowledged. Firstly, the number of studies included in this meta-analysis was limited. Secondly, there is relatively high heterogeneity in the calculation of peritumoral TLSs. Therefore, we explored potential sources of heterogeneity and suggested directions for future research. Thirdly, due to sample size limitations, we were unable to conduct subgroup analyses to explore the role of TLSs in different stages of HCC and other aspects. Fourthly, four studies are from a single country which introduces some kind of regional bias in the ultimate results of the meta-analysis.

## Conclusion

The existence of intratumoral TLSs is linked to enhanced RFS and reduced early recurrence in patients with HCC. Further exploration is required to understand the impact of peritumoral TLSs on the prognosis of HCC patients.

## Data availability statement

The original contributions presented in the study are included in the article/**Supplementary Material**. Further inquiries can be directed to the corresponding authors.

## Author contributions

LH: Conceptualization, Data curation, Formal analysis, Visualization, Writing – original draft. XL: Formal analysis, Writing – review & editing. CY: Visualization, Writing – review & editing. BZ: Data curation, Writing – review & editing. CD: Writing – review & editing. NJ: Writing – review & editing.
