# Supplementary material for: Prognostic value of tertiary lymphoid structures in hepatocellular carcinoma: a meta-analysis and systematic review
Source: Front Immunol. 2024 Jun 3;15:1390938. doi: 10.3389/fimmu.2024.1390938 (PMC11180782; doi:10.3389/fimmu.2024.1390938)

Supplementary material S1. Search strategy

| Databases | Search strategy |
| --- | --- |
| Pubmed | **#1 ((hepatocellular carcinoma[MeSH Terms]) OR (hepatocellular carcinoma)) OR (hcc)**  **#2 ((tertiary lymphoid structure) OR (tertiary lymphoid structures)) OR (TLS)**  **#3 #1 AND #2** |
| Embase | **#1** 'liver cell carcinoma'/exp OR 'hepatocellular carcinoma':ti,ab,kw OR hcc:ti,ab,kw  #2 'tertiary lymphoid structure'/exp OR 'tertiary lymphoid structure':ti,ab,kw OR 'tertiary lymphoid structures':ti,ab,kw OR tls:ti,ab,kw  **#3 #1 AND #2** |
| Cochrane Library | #1 MeSH descriptor: [Carcinoma, Hepatocellular]  #2 (hepatocellular carcinoma):ti,ab,kw OR (HCC):ti,ab,kw  #3 #1 OR #2  #4 **(tertiary lymphoid structure) :ti,ab,kw OR (tertiary lymphoid structures) :ti,ab,kw OR (TLS) :ti,ab,kw**  **#5 #3 AND #4** |
| Web of Science | **#1 ALL=(hepatocellular carcinoma) OR ALL=(hcc)**  **#2 ((ALL=(tertiary lymphoid structure)) OR ALL=(****tertiary lymphoid structures)) OR ALL=(TLS)** |

Supplementary material S2. Definition of TLS

| Study | Definiton of TLS |  |
| --- | --- | --- |
|  | Definition of intra-tumoral TLS |  |
| Zhang 2023 | The complete absence of TLS in the whole section was determined as TLS-, and the others were TLS+. |  |
| Jia 2021 | Samples with at least one occurrence of any form of iTLS (lymphoid aggregates, lymphoid follicles formation without germinal center and lymphoid follicles formation with germinal center) were categorized as the TLS+ group and the samples without any occurrence of iTLS were categorized as the TLS- group. |  |
| Li 2020 | The TLS were divided into three main grades: lymphoid aggregates, lymphoid follicles formation without germinal center and lymphoid follicles formation with germinal center. Tumors with at least one observable TLS were defined as intratumoral TLS+, whereas tumors without any observable TLS were defined as TLS-. |  |
| Calderaro 2019 | TLSs were classified as: i) Aggregates (Agg): vague, ill-defined clusters of lymphocytes; ii) Primary follicles (FL-I): round-shaped clusters of lymphocytes without germinal center formation and iii) Secondary follicles (FL-II): follicles with germinal center formation. Tumors with at least 1 intra-tumoral TLS were classified as TLS+, and tumors without any TLSs as TLS-. |  |
|  | Definition of peritumoral TLS |  |
| Zhang 2023 | Area: The peritumoral tissue was obtained at a distance of 1 cm from the tumor edge. Classification:TLS-, No aggregate in whole slide; TLS Low and TLS High distinguished by the cut-off of total area of immune aggregation. Cut-off value: The minimum p value method was used to further divide TLS+ into two groups, the TLS low group and the TLS high group. In our work, the cut-off values of peritumor is 109,941 pixels. |  |
| Wen 2022 | Area:The TLS we calculated was the density of TLS observed in the intertumoral and peritumoral 5 mm locations. Cut-off value: Cutoff points of TLS density were determined using the receiver operating characteristics (ROC) curve analysis. |  |
| Li 2021 | Area: The density of TLS was calculated as number/mm2 in peritumoral (5 mm from the infiltrative tumor border) regions. Cut-off value: The optimal threshold of pTLS density was identified by application of the receiver operating characteristics (ROC) curve. |  |
| Calderaro 2019 | Area: Areas of non-tumoral liver located less than 2 mm from tumor margins were excluded from the analysis. Cut-off value: Cases with a density superior to the median of the full series were classified as TLS NT+, and cases with a density under the median as TLS NT-. |  |
|  |  |  |

Supplementary material S3. NOS score for included studies

| Study | Selection | | | | Comparability | Outcome | | | Total score |
| --- | --- | --- | --- | --- | --- | --- | --- | --- | --- |
|  | Representativeness  of the  exposed cohort | Selection of  the non- exposed cohort | Ascertainment  of exposure | Demonstration  of outcome |  | Assessment of outcome | Follow-up was long enough | Adequacy  of follow up |  |
| Intratumor TLS |  |  |  |  |  |  |  |  |  |
| Zhang 2023 | * | * | * | * | * | * | * | * | 8 |
| Jia 2022 | * | * | * | * |  | * | * | * | 7 |
| Li 2020 | * | * | * | * | * | * | * | * | 8 |
| Calderaro 2019 | * | * | * | * | * | * | * | * | 8 |
| Peritumoral TLS |  |  |  |  |  |  |  |  |  |
| Zhang 2023 | * | * | * | * | * | * | * | * | 8 |
| Wen 2022 | * | * | * | * | * | * | * | * | 8 |
| Li 2021 | * | * | * | * |  | * | * | * | 7 |
|  |  |  |  |  |  |  |  |  |  |

Supplementary material S4. Sensitivity analysis for intratumoral TLSs. A, overall survival; B, recurrence-free survival; C, early recurrence; D, late recurrence.


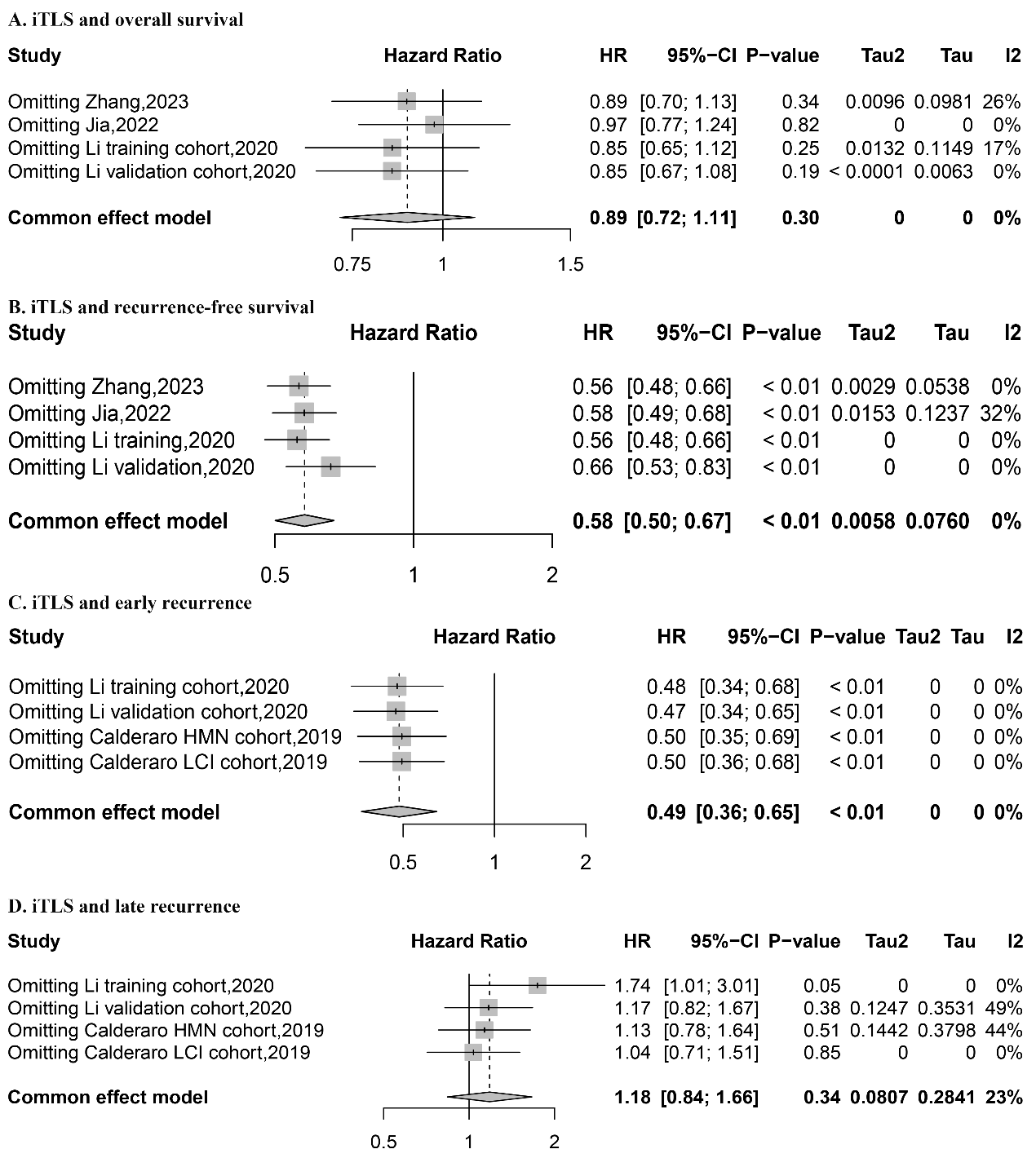


Supplementary material S5. Funnel plot of intratumoral TLSs. A, overall survival; B, recurrence-free survival; C, early recurrence; D, late recurrence.

A.


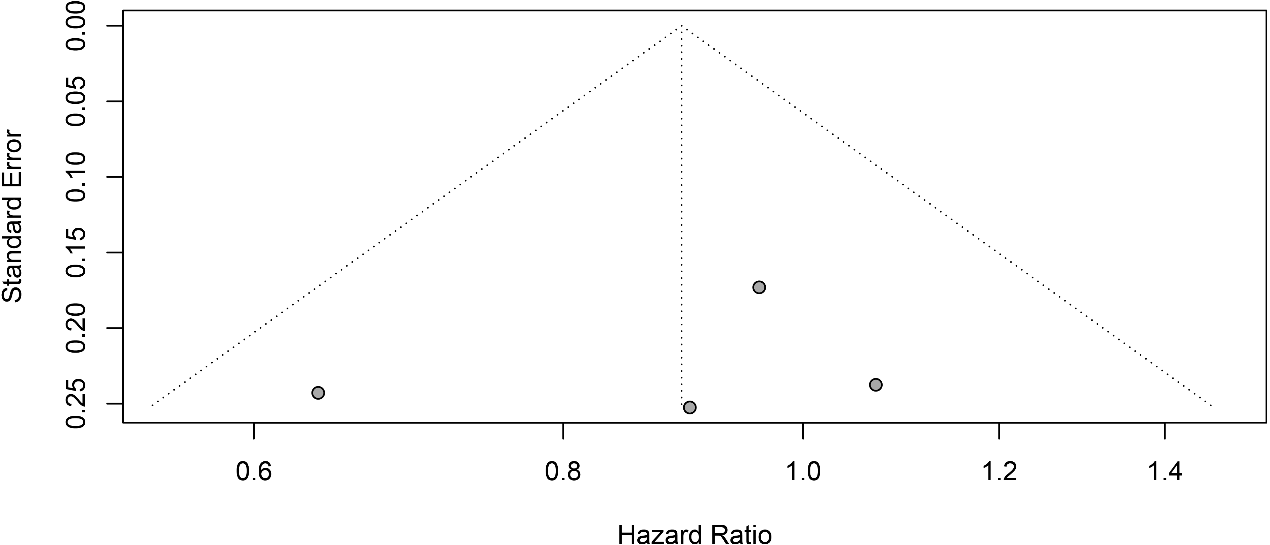


B.


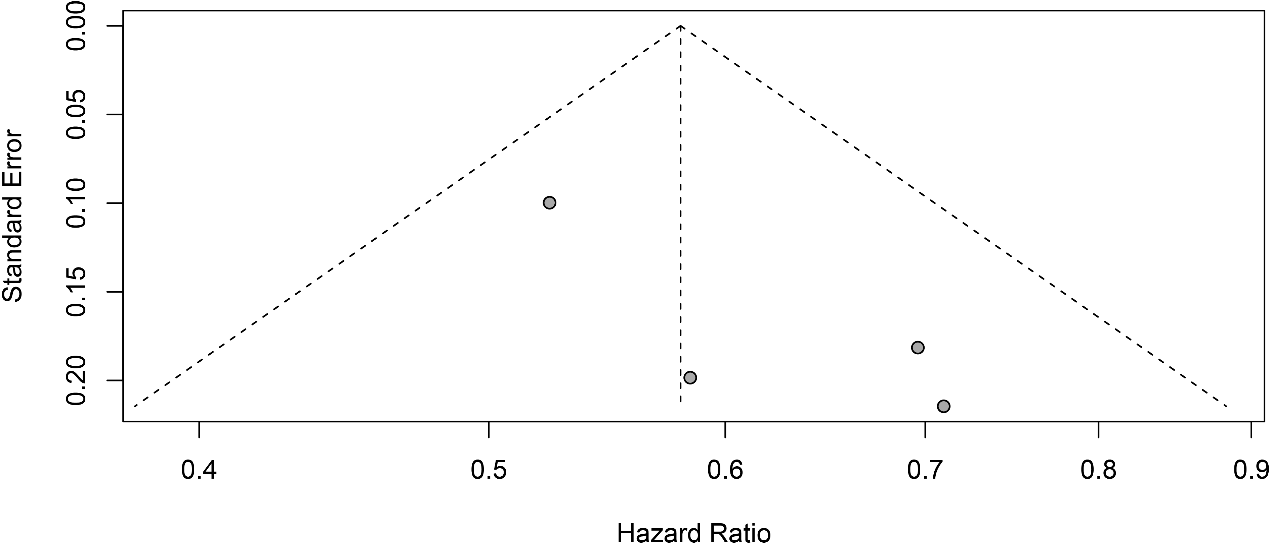


C.


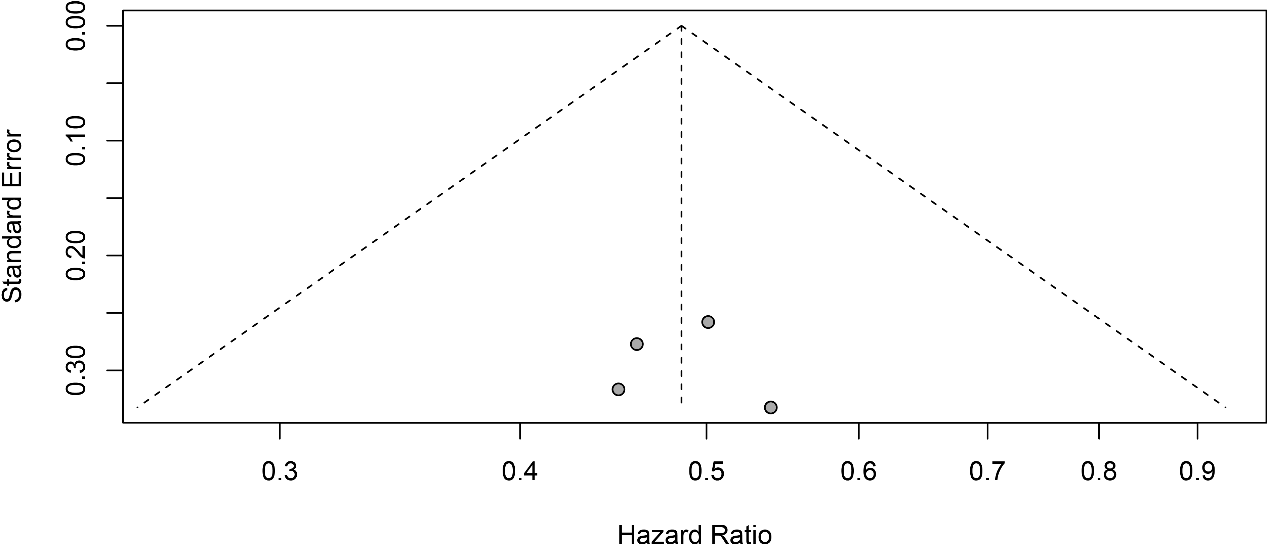


D.


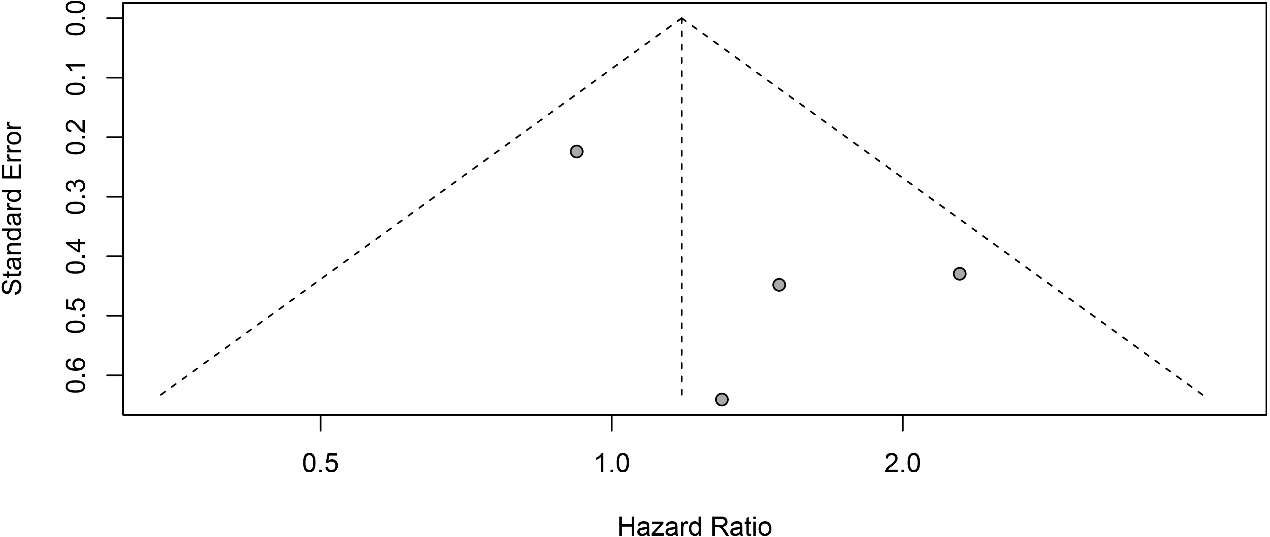


Supplementary material S6. Sensitivity analysis for peritumoral TLSs. A, overall survival; B, recurrence-free surviva


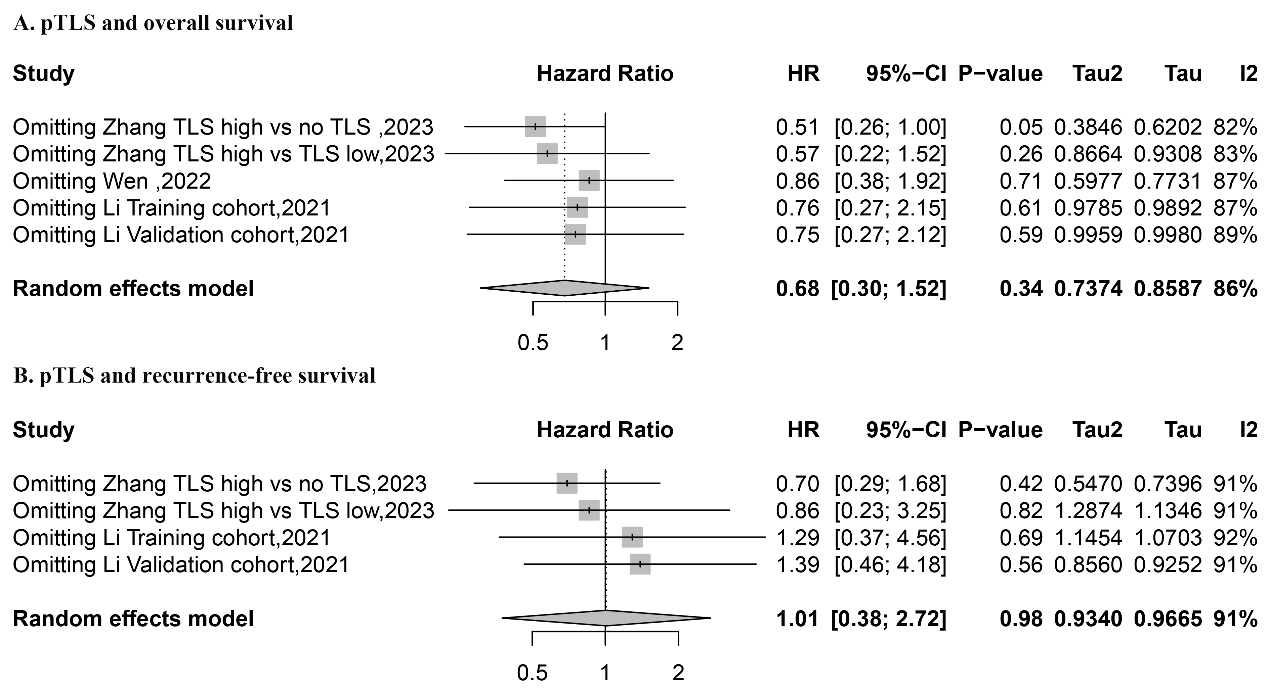


Supplementary material S7. Sensitivity analysis for peritumoral TLSs by omitting data from the article by Zhang *et al.*. A, overall survival; B, recurrence-free surviva


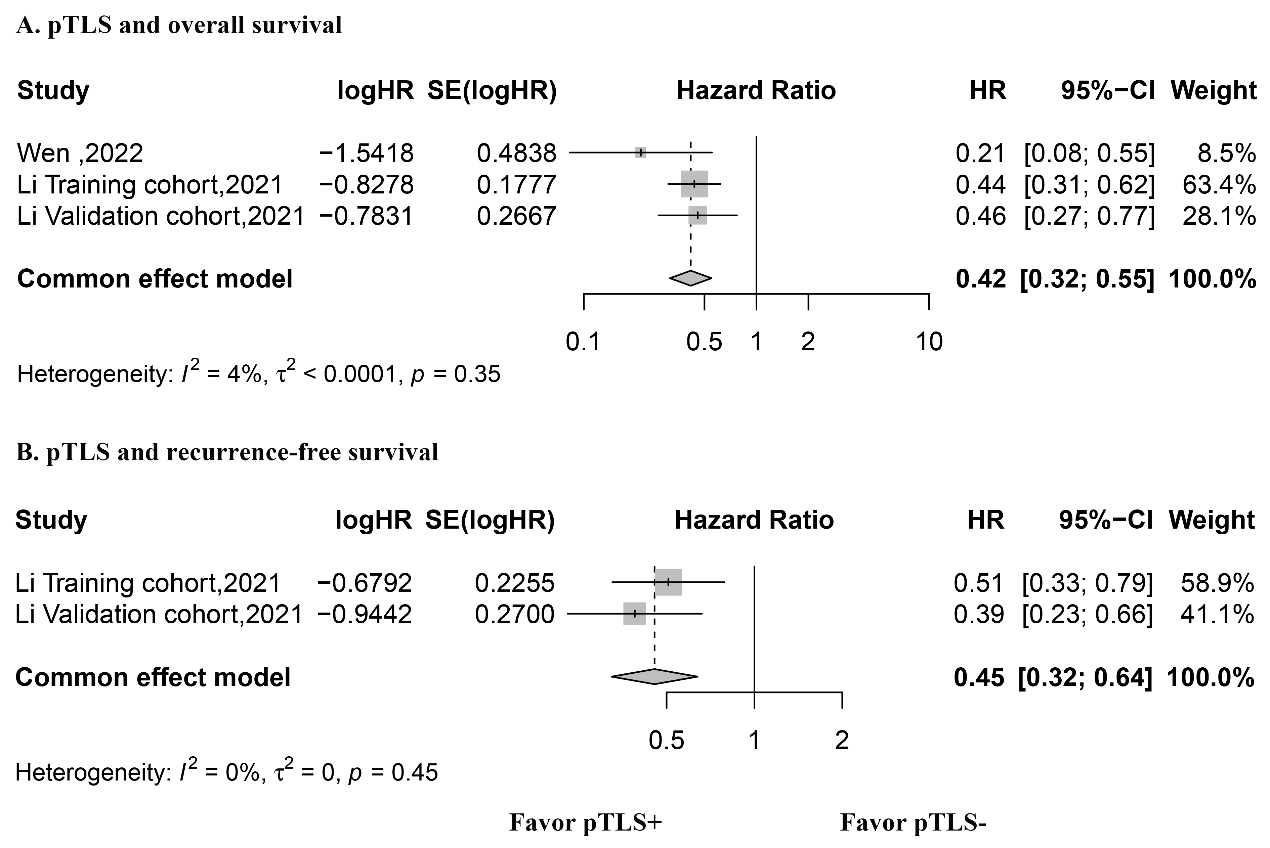


Supplementary material S8. Funnel plot of peritumoral TLSs. A, overall survival; B, recurrence-free survival.


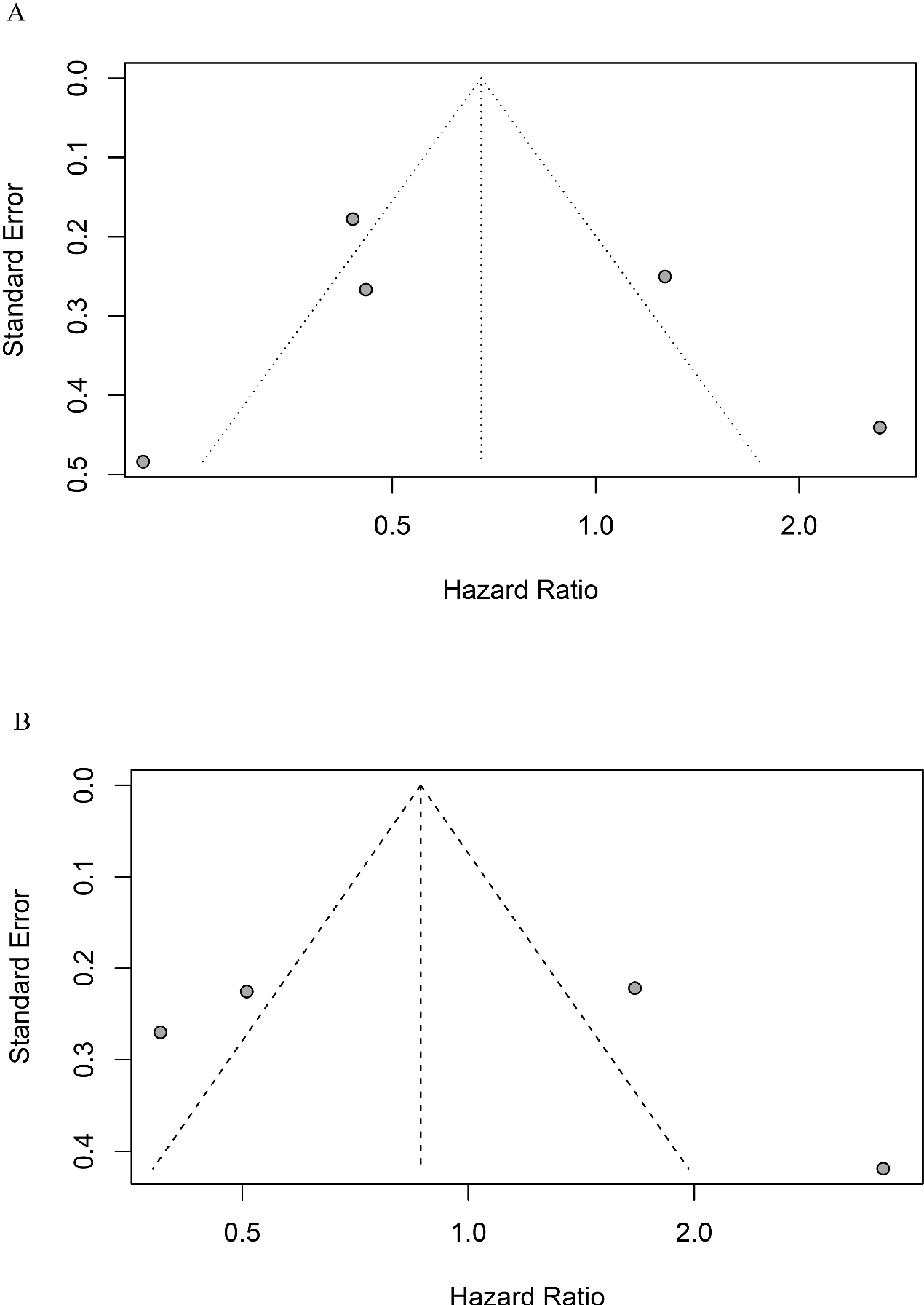

Supplement: Supplementary file 1 [file DataSheet_1.docx]
